# Study of correlations between protein peptide plane dynamics and side chain dynamics

**DOI:** 10.1371/journal.pone.0215141

**Published:** 2019-04-12

**Authors:** Yanzhen Hou, Jiaojiao Liu, Jianfeng He, Xubiao Peng, Antti J. Niemi

**Affiliations:** 1 School of Physics, Beijing Institute of Technology, Beijing 100081, P.R. China; 2 Center for Quantum Technology Research and School of Physics, Beijing Institute of Technology, Beijing 100081, P.R. China; 3 Laboratoire de Mathematiques et Physique Theorique CNRS UMR 6083, Fédération Denis Poisson, Université de Tours, Parc de Grandmont, F37200, Tours, France; 4 Nordita, Stockholm University, Roslagstullsbacken 23, SE-106 91 Stockholm, Sweden; 5 Laboratory of Physics of Living Matter, Far Eastern Federal University, 690950, Sukhanova 8, Vladivostok, Russia; University of Michigan, UNITED STATES

## Abstract

Protein dynamics is pivotal to biological processes. However, experiments are very demanding and difficult to perform, and all-atom molecular dynamics simulations can still not provide all the answers. This motivates us to analyze protein dynamics in terms of different reduced coordinate representations. We then need to resolve how to reconstruct the full all-atom dynamics from its coarse grained approximation. Accordingly we scrutinize all-atom molecular dynamics trajectories in terms of crystallographic Protein Data Bank (PDB) structures, and inquire to what extent is it possible to predict the *dynamics* of side chain C*β* atoms in terms of the *static* properties of backbone C*α* and O atoms. Here we find that simulated C*β* dynamics at near physiological conditions can be reconstructed with very high precision, using the knowledge of the crystallographic backbone C*α* and O positions. The precision we can reach with our PDB-based *Statistical Method* reconstruction exceeds that of popular all-atom reconstruction methods such as *Remo* and *Pulchra*, and is fully comparable with the precision of the highly elaborate *Scwrl4* all-atom reconstruction method that we have enhanced with the knowledge of the backbone C*α* and O atom positions. We then conclude that in a dynamical protein that moves around at physiological conditions, the relative positions of its C*β* atoms with respect to the backbone C*α* and O atoms, deviate very little from their relative positions in static crystallographic PDB structures. This proposes that the dynamics of a biologically active protein could remain subject to very similar, stringent stereochemical constraints that dictate the structure of a folded crystallographic protein. Thus, our results provide a strong impetus to the development of coarse grained techniques that are based on reduced coordinate representations.

## Introduction

The C*α* atoms are located at the branch points of a protein, they connect the backbone and the side chains. As a consequence their positions are subject to relatively tight stereochemical constraints. Indeed, in the case of static crystallographic proteins, the all-atom structure can be often determined with a good precision from the knowledge of the C*α* positions [1–9]. This motivates the so-called C*α* trace problem where the goal is to construct an accurate all-atom model of a crystallographic folded protein structure solely from the knowledge of the positions of its C*α* atoms [10–15].

Protein dynamics is pivotal to biological function and many proteins are presumed to be flexible under physiological conditions. Unlike in the case of static crystallographic protein structures, the relative positions of the various atoms can then become variable. There is no *a priori* reason why the dynamical all-atom structure could be determined solely from the knowledge of the C*α* backbone. Instead the peptide planes and the side chains are expected to twist and bend with respect to the C*α* backbone, presumably in a complicated fashion. However, high precision experiments in the case of a dynamical protein are very difficult to perform, and our knowledge of atomic level protein dynamics remains limited [16–19]. Presently, the best sources of information are all-atom molecular dynamics simulations. These are probably best exemplified by the very long *Anton* folding trajectories [20, 21].

In [22] a dynamical variant of the C*α* trace problem is addressed. For example, two all-atom *Anton* trajectories were analyzed in detail, with focus on the peptide plane O atoms and side chain C*β* atoms: The O atom is the only heavy peptide plane atom that is not covalently connected to the C*α* and the C*β* defines the direction of the side chain. Thus, these two atoms have a particularly important structural information content. Unexpectedly, it was found that the motions of the peptide plane O atoms and the side chain C*β* atoms could be reconstructed with high precision solely from the knowledge of the C*α* atom dynamics, using a statistical analysis of *static* crystallographic protein structures in Protein Data Bank (PDB) [23]. The results suggest that in an isolated dynamical protein, at near physiological conditions, the motions of its O and C*β* atoms remain strongly slaved to the C*α* dynamics, to the extent that they are more or less only subject to modest thermal fluctuations. If this is indeed the case, the problem of protein dynamics at physiological conditions could be much simpler than expected: If the motions of different atoms are strongly correlated and in a systematic manner, many aspects of protein dynamics could be modelled by an effective coarse grained energy function, formulated in terms of a reduced sets of coordinates that describe only the C*α* atoms [24–26] or a group of atoms including the C*α* as effective interaction centers [27–29].

Here we present an analysis of simulated all-atom protein dynamics in terms of reduced coordinate sets. We focus on the interrelations between the C*α*, peptide plane O and side chain C*β* atoms. This choice is partly motivated by a series of articles [30–32] that propose to investigate the interrelations between the C*α*, the peptide plane O and the side chain C*β* atoms in a Hamiltonian context. Specifically, we inquire how accurately can the dynamics of the C*β* atoms be determined from the knowledge of the backbone C*α* and O atom dynamics, using solely a statistical analysis of *static* crystallographic PDB structures. We select these three groups of atoms for their pivotal structural role: Alongside the C*α* atoms the peptide plane O atoms and side chain C*β* atoms are both structurally highly important. The O atoms are the only backbone heavy atoms that have no covalent bond with C*α* atoms, but they have many very important formative functions. For example, in combination with the peptide plane H atoms they forge and stabilize regular secondary structures including *α*-helices and *β*-strands. Similarly, the C*β* atoms determine the orientations of the side chains, strongly affecting the positions of all the higher level side chain atoms. Indeed, in the case of crystallographic protein structures, the knowledge of the peptide planes and the C*β* atom positions is quite sufficient to determine the other side chain atom positions in terms of rotamer libraries [33–37]. This is amply demonstrated by the success of reconstruction programs such as *Pulchra* [13], *Remo* [14] and *Scwrl4* [15].

Our aim is to understand how protein dynamics could be modelled in an effective manner. For this we propose to investigate and develop different coordinate descriptions that can adapt to the dynamics. Previously, several different coordinate systems have been proposed in the literature. Particularly notable are the Ramachandran angles [38, 39], widely used to validate crystallographic protein structures. But these angles provide only local information, they are not sufficient to describe the three dimensional shape, and thus, the dynamics, of a protein [40]. For this alternative coordinate systems have been developed, often starting by envisioning the C*α* backbone as a piecewise linear polygonal chain for which the C*α* coordinates $r_{i}^{\alpha }(i=1,\ldots ,N)$ define vertices; here *N* is the number of C*α* atoms and we start the indexing from the N terminus. As a linear chain the C*α* backbone can be framed, and one way is to employ a discrete version of the Frenet frames [41, 42]. The Frenet frames are purely geometric, they do not make any reference to the other heavy atoms of the protein. Nevertheless, discrete Frenet frames provide a very convenient description of a crystallographic protein structure, as it turns out that the C*β* and peptide plane heavy atoms are all highly localized in terms of Frenet frames, quite independently of the proteins amino acid content.

Other purely geometric ways to frame the C*α* backbone can also be introduced. An example is the parallel transport (Bishop) framing [43]. But in terms of parallel transport frames the C*β* and peptide plane atoms fail to localize [41]. Thus, these frames do not seem to be very convenient, in the study of protein structure. We also note that reconstruction programs such as *Pulchra*, *Remo* and others, commonly use their own, specially designed framings that are built on the C*α* backbone geometry.

Besides geometry based frames, proteins can also be framed using their structure; the Ramachandran angles are an example. In particular, the combination of C*α* and C*β* atoms defines such a structure based framing [41, 42]. It employs the covalent bond that connects these two atoms, in addition of the virtual bonds that connect neighboring C*α* atoms, to define the local frame. Such a C*β* framing adapts well to all amino acids except for glycine that has no C*β* atom. Since the C*β* atoms are highly inert in the discrete Frenet frames, such a C*α*C*β* based framing yields a description of the protein structure that is very similar to the discrete Frenet framing description.

Here we develop an alternative structure based framing, using the peptide planes: The normal vector of a peptide plane, in combination with the virtual bonds between the C*α* atoms, can be employed to define such a framing. Our aim is to study how such a peptide plane based framing describes protein dynamics. For this we analyze data from two extended *Anton* simulations [20] that are performed with *Charmm22^⋆^* force field [21]. The trajectories that we consider are the villin and the ww-domain, reported in [20]. Villin is *α*-helical and ww-domain is *β*-stranded in a folded state. The *Anton* simulations observe several transitions between structures that are (apparently) unfolded and that are (apparently) folded. Thus, the combination of these two trajectories cover many local structures, with all the biologically relevant amino acids appearing, except CYS with its unique potential to form sulphur bridges. Moreover, the villin in [20] involves a NLE mutant and the HIS in [20] is protonated. Thus, our analysis includes the effects of mutations and pH variations. All this ensures that there is a good diversity of dynamical details for us to analyze, in terms of these two trajectories.

The length of the villin trajectory is 120*μ*s, and we have selected every 20*th* simulated structure for our prediction analysis, for a total of 31395 structures. The length of the ww-domain trajectory is 651 *μ*s and we have chosen every 40*th* simulated structure for our prediction analysis, for a total of 60814 structures. We use the coordinates of the backbone C*α* and O atoms along the *Anton* trajectories, to try and reconstruct the coordinates of the C*β* atoms using a variety of methods. We then compare the results with the C*β* coordinates along the *Anton* trajectories. Besides a C*α*-Frenet frame based reconstruction [22], and the peptide plane based *Statistical Method* reconstruction that we develop here, we also analyze the results that we obtain using commonly available all-atom structural reconstruction programs *Remo* and *Scwrl4*; here we use and enhanced variant of *Scwrl4* that accounts for the positions of the backbone C*α* and O atoms. We have also performed the analyses using *Pulchra*, with results that comparable to those we obtain with *Remo*. For clarity of representation, we do not present the *Pulchra* results explicitely.

Finally, we remind that the *Anton* simulations provide us with *dynamical* data at physiologically relevant temperature values while all the reconstruction algoritms that we use, are based on *static* crystallographic structures that are commonly measured at very low (liquid nitrogen) temperatures. Thus, our results should also reveal how the structure deforms due to temperature effects in addition of dynamics.

## Methods

### Framing a protein backbone

#### Purely geometric discrete Frenet frames

The discrete Frenet frames are purely geometric frames, they are built solely in terms of the C*α* atoms of a protein backbone. Thus, these frames can be useful *e.g*. for reconstruction of the atomic structure when only the C*α* atom positions are known. We define the discrete Frenet frames as follows: We assign to each C*α* position **r**_*i*_ an orthonormal triplet (**n**_*i*_, **b**_*i*_, **t**_*i*_) [41, 42]. We construct these vectors by first identifying **t**_*i*_ with the vector that points from the center of the *i*^*th*^ C*α* towards the center of the (*i* + 1)^*st*^ C*α*,

$$\begin{array}{c} t_{i}\;=\;\frac{r_{i+1}-r_{i}}{|r_{i+1}-r_{i}|} \end{array}$$ (1)

The binormal vector is normal to the plane formed by three consecutive C*α* carbons,

$$\begin{array}{c} \;b_{i}\;=\;\frac{t_{i-1}\times t_{i}}{|t_{i-1}\times t_{i}|} \end{array}$$ (2)

and the normal vector is

$$\begin{array}{c} \;n_{i}\;=\;b_{i}\times t_{i} \end{array}$$ (3)

**Fig 1:**
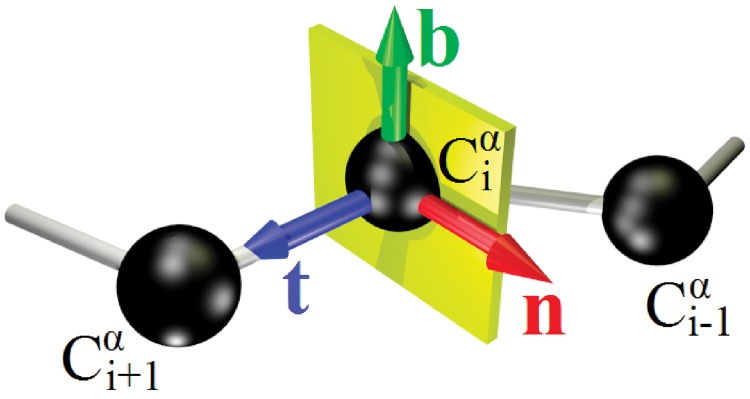
Discrete Frenet frame vectors (1), (2) and (3).

For visualization we use a color coding with **n** ∼ *x* ∼ *red* (*r*), **b** ∼ *y* ∼ *green* (*g*) and **t** ∼ *z* ∼ *blue* (*b*) in terms of a right-handed Cartesian (*xyz*)∼(*rgb*) coordinate system; see Fig 1.

We use the discrete Frenet frame vectors to define the (virtual) C*α* backbone bond (*κ*) and torsion (*τ*) angles as follows,

$$\begin{array}{c} \begin{array}{c} \kappa_{i}=\arccos\left(t_{i+1}\cdot t_{i}\right)\\ \tau_{i}=\text{sign}\left(b_{i+1}\cdot n_{i}\right)\arccos\left(b_{i+1}\cdot b_{i}\right) \end{array} \end{array}$$ (4)

**Fig 2:**
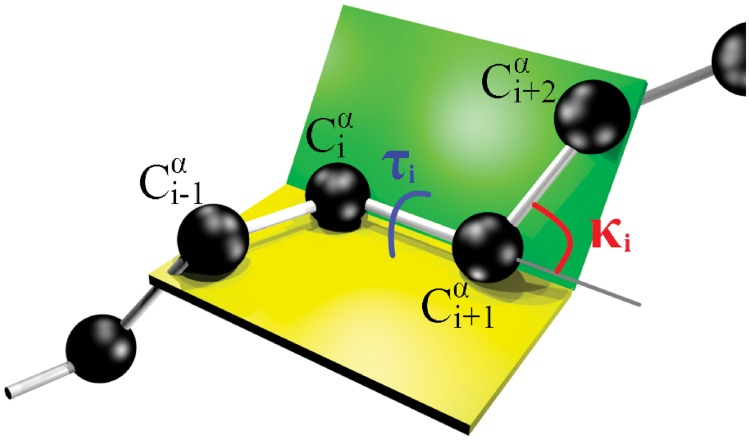
Definition of (virtual) C*α* backbone bond (*κ*) and torsion (*τ*) angles.

These angles are shown in Fig 2 We identify them as the canonical latitude (*κ*) and longitude (*τ*) angles on the surface of a unit radius (Frenet) sphere $S_{\alpha }^{2}$ that is centered at the C*α* atom; the sphere is oriented so that the north pole is in the direction *κ* = 0. Thus, the discrete Frenet frame vector **t** points to the direction of the north pole; it coincides with the *z*-direction in a C*α* centered Cartesian coordinate system. The torsion angle *τ* coincides with the longitude angle and takes values *τ* ∈ [−*π*, *π*), increasing in the counterclockwise direction around the positive *z*-axis *i.e*. around vector **t**. The great circle *τ* = 0 passes through the north pole and the tip of the normal vector **n** that lies at the equator.

#### Structure based peptide plane C*α*O frames

When in addition of the C*α* atom positions also *e.g*. the peptide plane O atom positions are all known, we can proceed and frame the protein backbone in a structure dependent fashion: The C*α* atoms C*α*(*i*) and C*α*(*i* + 1) that are located at the *i*^*th*^ and (*i* + 1)^*st*^ sites define two corners of the (virtual) peptide plane. The third corner coincides with the *i*^*th*^ O atom. Accordingly, when we continue to denote the C*α*(*i*) coordinates by **r**_*i*_ and denote the O(*i*) coordinates by $r_{O_{i}}$, the O_*i*_ centered C*α*O frames are defined by the following right-handed orthonormal triplet ($\hat{u},\hat{v},\hat{w})∼(xyz)∼(rgb)$

$$\begin{array}{c} {\hat{u}}_{i}=\frac{r_{i+1}-r_{i}}{|r_{i+1}-r_{i}|} \end{array}$$ (5)

$$\begin{array}{c} {\hat{w}}_{i}=\frac{r_{i+1}-r_{i}}{|r_{i+1}-r_{i}|}\times \frac{r_{O_{i}}-r_{i}}{|r_{O_{i}}-r_{i}|} \end{array}$$ (6)

$$\begin{array}{c} {\hat{v}}_{i}={\hat{w}}_{i}\times {\hat{u}}_{i} \end{array}$$ (7)

**Fig 3:**
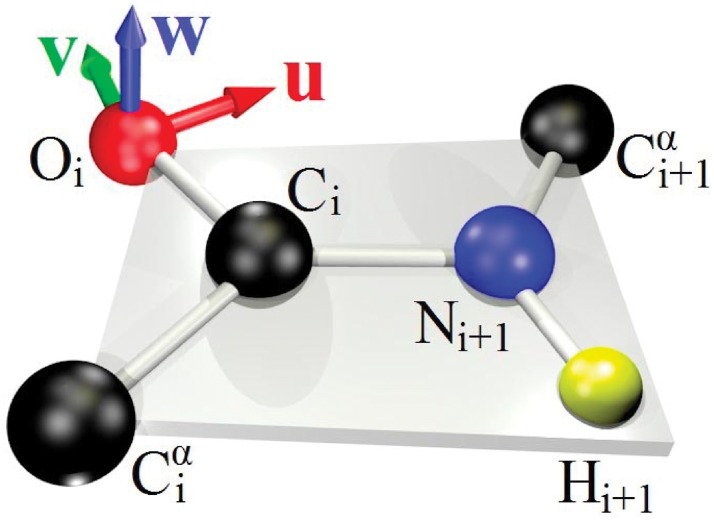
The C*α*O frames are centered at the O atom of the peptide plane.

**Fig 4:**
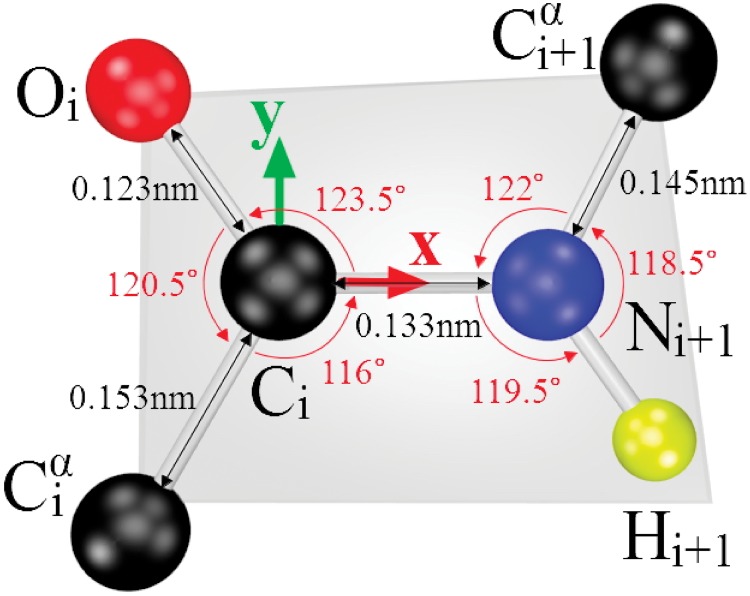
The detailed structure of the ideal peptide plane (IPP) that we use.

In Fig 3 we show these frames. In our reconstruction we shall assume that the peptide planes are ideal. The vector ${\hat{w}}_{i}$ is then a normal vector of the (ideal) peptide plane and we can estimate the coordinates of the remaining peptide plane atoms C_*i*_, N_*i*+1_ and H_*i*+1_, starting from the ideal peptide plane (IPP) geometry that we display in Fig 4.

### Visualizing a protein structure

#### C*α* atom visualization

The PDB data pool that we use is the same as used in [22]; see also [40]. It is updated continuously, to consist of all contemporary crystallographic protein structures that have been measured with better than 1.0 Å resolution. We trust that such ultra high resolution structures have been subjected to (at most) very minimal refinement.

**Fig 5:**
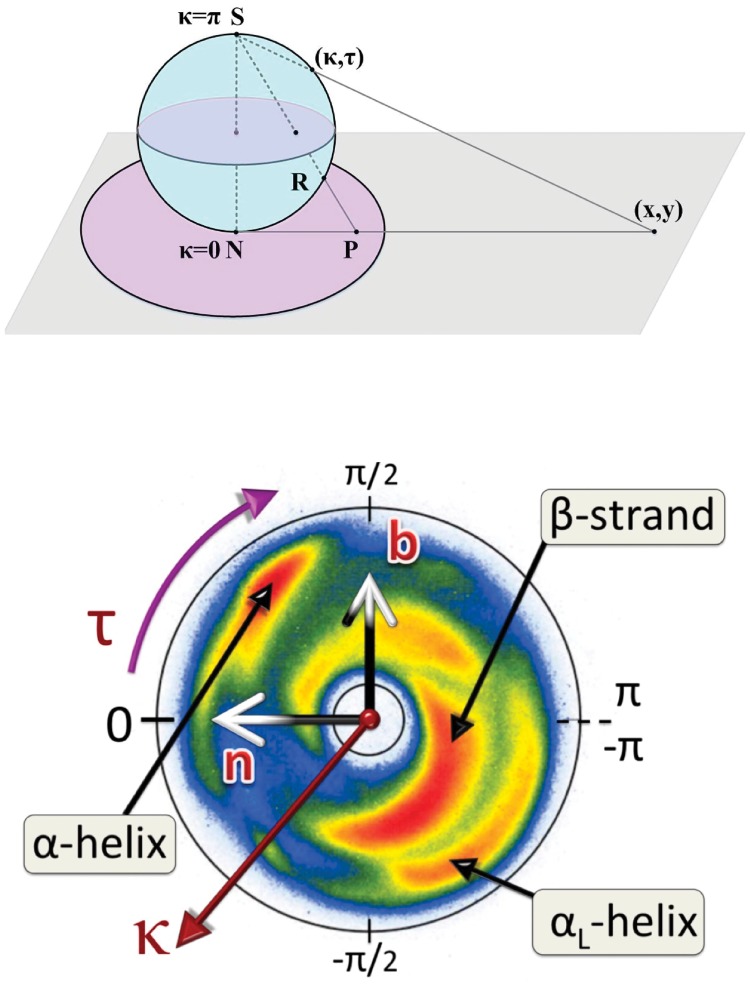
Top: Stereographic projection of two sphere onto plane, with the projection taken from the south pole. Bottom: Statistical distribution of all C*α* atoms in our pool of PDB structures, on stereographically projected sphere.

For visualization purposes, we use the statistical distribution of the C*α* backbone bond and torsion angles on a stereographically projected two-sphere $S^{2}$, shown in Fig 5 and constructed as follows: Let $S_{\alpha }^{2}\left(i\right)$ be a (unit radius) sphere that is centered at the *i*^*th*^ C*α* atom of a given PDB structure. The vector **t**_*i*_ then has its tail at the location of C*α*(*i*) and its head lies at the north pole of $S_{\alpha }^{2}\left(i\right)$, pointing towards the C*α*(*i* + 1) atom. The following atom C*α*(*i* + 2) is located similarly, in the direction of the discrete Frenet frame vector **t**_*i*+1_ that points from the center of $S_{\alpha }^{2}\left(i+1\right)$ towards its north pole. We parallel transport **t**_*i*+1_, with no rotation, until its tail coincides with the origin of $S_{\alpha }^{2}\left(i\right)$. Let (*κ*_*i*_, *τ*_*i*_) be the discrete Frenet frame coordinates of the parallel transported head of **t**_*i*+1_ on the surface of $S_{\alpha }^{2}\left(i\right)$. These coordinates describe how a miniature observer at the position of C*α*(*i*) atom, with head oriented towards the north pole of $S_{\alpha }^{2}\left(i\right)$, finds the backbone twisting and bending when she proceeds along the chain to the position of C*α*(*i* + 1).

After we repeat this construction for all the C*α* atoms of all PDB structures in our PDB data pool, we obtain a statistical distribution of coordinates (*κ*, *τ*). We visualize this distribution by projecting the sphere $S_{\alpha }^{2}$ stereographically onto the complex plane from the south pole as shown in Fig 5. The projection is defined by

$$\begin{array}{c} x+iy=\tan(\frac{\kappa }{2})e^{i\tau } \end{array}$$

In Fig 5 we also show the (*κ*, *τ*) distribution of all the C*α* atom coordinates in our PDB data set, on the stereographically projected Frenet sphere. The distribution is largely concentrated inside an annulus, with inner circle *κ*_*in*_ ≈ 1 and outer circle *κ*_*out*_ ≈ *π*/2. In the Figure we have identified the regular secondary structure regions corresponding to *α*-helices, *β*-strands and left-handed *α*-helices.

#### Visualization of peptide plane O atoms and side chain C*β* atoms

**Fig 6:**
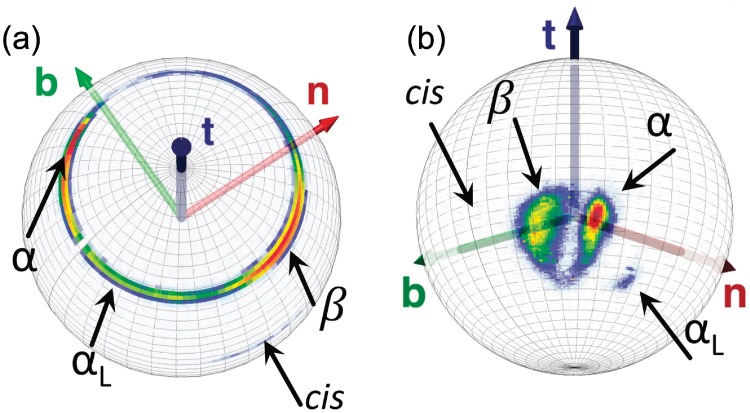
Fig a): The distribution of peptide plane O atom coordinates (*ϑ*, *ψ*) in our PDB pool of structures on the surface of the Frenet sphere $S_{\alpha }^{2}$. Fig b): The distribution of C*β* atom coordinates (*θ*, *ϕ*) in our PDB pool of structures on the surface of the Frenet sphere $S_{\alpha }^{2}$. In both Figures we have identified the *α*-helices (*α*), *β*-strands (*β*), left-handed *α*-helices (*α*_*L*_) and *cis*-peptide planes (*cis*).

To visualize the peptide plane O atom and side chain C*β* atom distributions in our PDB data set, we use the C*α* centered Frenet spheres $S_{\alpha }^{2}$ but with no stereographic projection. We depict the direction of these atoms on the surface of $S_{\alpha }^{2}$, exactly as they are seen by a discrete Frenet frame observer who stands at the position of the C*α* atom. In Fig 6 we show the ensuing statistical distributions that we obtain for the O and C*β* atoms in our statistical pool of PDB structures. Again, the major regular secondary structures have been identified.

### The Statistical Method reconstruction algorithm

Our *Statistical Method* reconstruction algorithm is an extension of the algorithm developed in [22]. It builds on the C*α*O framing, it aims to use the known positions of C*α* and O atoms to predict the positions of the remaining atoms along a protein chain. As an example, we here predict the spherical coordinates (*θ*, *ϕ*) *i.e*. directions of C*β* atoms on the surface of a proper two-sphere $S^{2}$, using the knowledge of the C*α* coordinates (*κ*, *τ*) and O coordinates that we denote (*ϑ*, *ψ*) in the sequel.

We do not address fluctuations in the covalent bond lengths and other distance scales. By construction, any reconstruction that builds on crystallographic data can only reproduce (at most) very long time scale average values of these quantities. Besides, our analyses confirm that the radial distance variations in *Anton* trajectories are minor in comparison to the directional (angular) variations. Thus, for the C*α*-C*β* covalent bond length we use the *Anton* average value 1.55 Å and for the other distances we refer to Fig 4.

We start as follows [22]: We divide the statistical distribution of our PDB data shown in Fig 5 (bottom) into 60 equal sectors with angular size Δ*τ* = *π*/30 radians. We then divide each of these sectors into two radial sets, one corresponding to bond angle values *κ* > 1.2 (rad) and the other to *κ* ≤ 1.2 (rad): The value *κ* = 1.2 divides the annulus in Fig 5 (roughly) into two annuli corresponding to *α*-helix-like (*κ* > 1.2) and *β*-strand-like (*κ* < 1.2) structures.

Next, we observe that the statistical distribution of peptide plane O atoms shown in Fig 6 (top) forms a very narrow circular strip with latitude *ϑ* ≈ *π*/4. Accordingly, we further divide the PDB data in terms of the O atom coordinate values, by longitudinally dividing the statistical O distribution of Fig 6 into 90 equal length Δ*ψ* segments with length Δ*ψ* = *π*/45.

We use our division of PDB structures according to the (*κ*, *τ*, *ψ*) values, to predict the C*β* coordinates (*θ*, *ϕ*) of a given structure as follows:

Step 1 We start from the known *τ*_*i*_ value of the given C*α*(*i*) atom, to select the pertinent sector *τ*_*i*_ ∈ Δ*τ*. We then use the *κ*_*i*_ value of C*α*(*i*) together with *κ*_*i*+1_ of the C*α*(*i* + 1) atom, to further assign C*α*(*i*) to one of the four sets
  $$\begin{array}{c} \begin{array}{c} \text{Set}\;\Delta \kappa_{1}:\;\;\;\;\;\;\;\;\kappa_{i}\;<\;1.2\;\;\;\;\&\;\;\;\;\kappa_{i+1}\;<\;1.2\\ \text{Set}\;\Delta \kappa_{2}:\;\;\;\;\;\;\;\;\kappa_{i}\;<\;1.2\;\;\;\;\&\;\;\;\;\kappa_{i+1}\;\geq \;1.2\\ \text{Set}\;\Delta \kappa_{3}:\;\;\;\;\;\;\;\;\kappa_{i}\;\geq \;1.2\;\;\;\;\&\;\;\;\;\kappa_{i+1}\;<\;1.2\\ \text{Set}\;\Delta \kappa_{4}:\;\;\;\;\;\;\;\;\kappa_{i}\;\geq \;1.2\;\;\;\;\&\;\;\;\;\kappa_{i+1}\;\geq \;1.2 \end{array} \end{array}$$
  We then use the *ψ*_*i*_ value of O(*i*) to select the Δ*ψ* segment. These steps identify a subset of [Δ*κ*;Δ*τ*;Δ*ψ*] to which we putatively assign the C*β*(*i*) atom.
Step 2 We proceed to consider those PDB structures for which the (*κ*, *τ*, *ψ*) values are in the same subset [Δ*κ*;Δ*τ*;Δ*ψ*]. Among these we search for a PDB structure that has equal amino acids at two consecutive sites, as the C*β* atom of interest.
  - If we find only one such matching PDB structure we use the coordinates of its C*β* atom for our prediction.
  - If we have more than one pair of matching amino acids in the PDB subset, we use the average values of the ensuing C*β* coordinates for our prediction.
  - If there are no PDB entries with matching amino acid pairs, we use the average value of C*β* coordinates (*θ*, *ϕ*) of all PDB structures in the given subset [Δ*κ*;Δ*τ*;Δ*ψ*] for our prediction.
  - Finally, if the PDB subset [Δ*κ*;Δ*τ*;Δ*ψ*] has no entries, we use a neighboring subset for our prediction. In selecting the neighboring subset, we move clockwise in along the strip of O atoms, then clockwise in terms of the torsion angle.

In Step 2, the average latitude value *κ*_*ave*_ is simply

$$\begin{array}{c} \kappa_{ave}\;=\;\frac{1}{N}\sum_{i=k}^{N}\kappa_{k} \end{array}$$

where the summation is over all elements in the given subset [Δ*κ*;Δ*τ*;Δ*ψ*]. For the average longitude value *τ*_*ave*_ we first define

$$\begin{array}{c} X\;=\;\frac{1}{N}\sum_{i=k}^{N}\cos\tau_{k}\;\;\;\;\;\&\;\;\;\;\;Y\;=\;\frac{1}{N}\sum_{i=k}^{N}\sin\tau_{k} \end{array}$$

and

$$\begin{array}{c} R=\sqrt{X^{2}+Y^{2}} \end{array}$$

The average value is then obtained from

$$\begin{array}{c} \cos\tau_{ave}\;=\;\frac{X}{R}\;\;\;\;\;\&\;\;\;\;\;\sin\tau_{ave}\;=\;\frac{Y}{R} \end{array}$$

We compute the average longitude value *ψ*_*ave*_ in the same way.

The present *Statistical Method* reconstruction algorithm is extremely simple and computationally very fast, in fact much faster than any of the other reconstruction programs that we have considered even though we have not optimized the search algorithm, but use a straightforward MatLab code. We note that the sizes of the subsets Δ*κ*, Δ*τ*, Δ*ψ* can be changed and optimized, the choice we have made here is only to exemplify the method.

## Results

We report on results that we have obtained using our *Statistical Method* reconstruction algorithm, and the publicly available reconstruction algorithms *Remo* and *Scwrl4*. Since *Scwrl4* requires that the peptide planes are known, we first construct the peptide planes using the C*α* and O atom coordinates from the *Anton* simulation. We then estimate the positions of peptide plane C, N and H atoms using the ideal peptide plane structure shown in Fig 4. Accordingly we denote this enhanced variant *ScwrIPP* in the sequel. The present *Statistical Method* reconstruction algorithm will be denoted *Stat* in the sequel. Note that we do not use the full *Anton* peptide planes in *ScwrIPP*. This is so that the information content in *ScwrIPP* and *Stat* are comparable.

### Description of Anton data distributions

We start by displaying the various data distributions in the *Anton* simulations; for the bond and torsion angles of the C*α* backbone, for the peptide plane structures and for the C*β* atoms on the surface of a two sphere.

#### Bond and torsion angle distributions in Anton simulations

**Fig 7:**
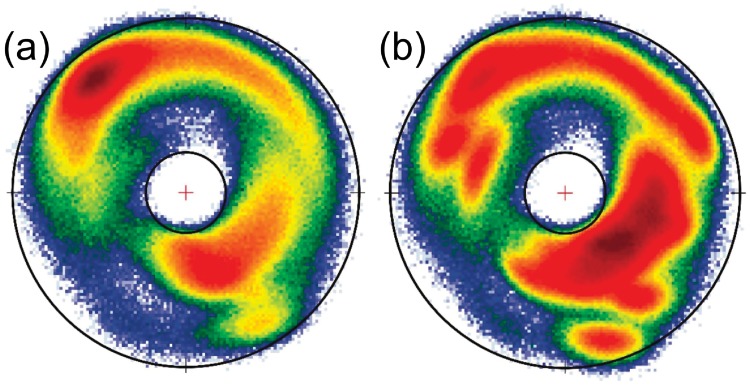
a) Statistical distribution of C*α* atoms in villin structures of *Anton*. b) Statistical distribution of C*α* atoms in ww-domain structures of *Anton*.

In Fig 7 we show the distributions of the C*α* bond and torsion angles on the stereographically projected two sphere of Fig 5, for the villin and ww-domain trajectories in the *Anton* data.

Both distributions in Fig 7 are somewhat different from the PDB distribution in Fig 5. Specifically, the villin distribution is largely concentrated in the vicinity of the *α*-helical region while the ww-domain distribution has a wider spread. The data distributions in Fig 7 both confirm that the *Anton* data has a substantial dynamical content.

#### Peptide plane structure in Anton simulations

**Fig 8:**
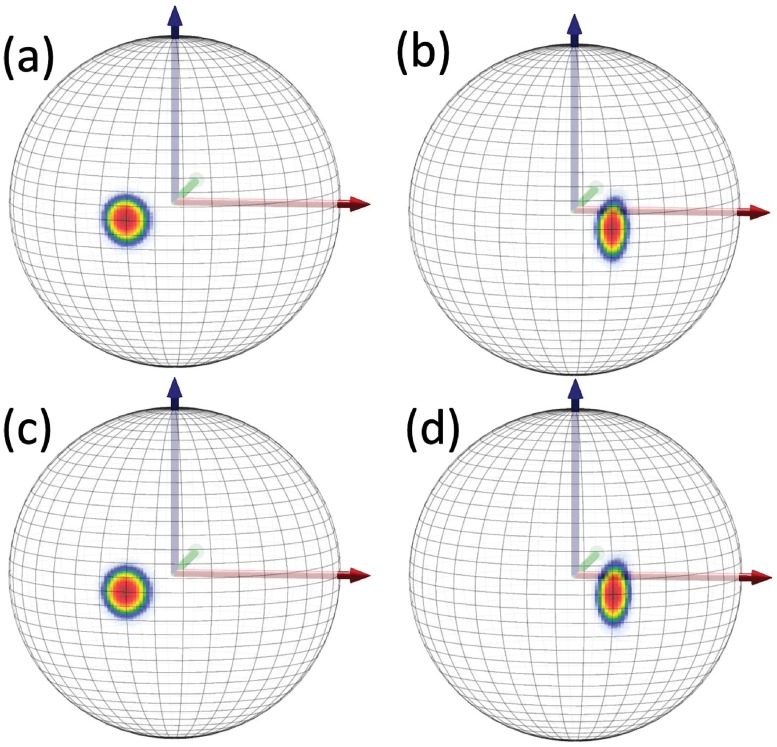
Statistical distribution of peptide plane C(*i*) and N(*i* + 1) atoms in *Anton* simulations, as seen in the O(*i*) centered C*α*O frames. a) C(*i*) in villin, b) N(*i* + 1) in villin, c) C(*i*) in ww-domain, d) N(*i* + 1) in ww-domain.

We proceed to confirm that the ideal, crystallographic peptide plane structure of Fig 4 is indeed preserved, in the course of *Anton* simulations. For this we plot the statistical distributions of the C(*i*) and N(*i* + 1) directions as seen in the O(*i*) centered C*α*O frames, in the *Anton* trajectories of villin and ww-domain. The results are shown in Fig 8: We observe that all four distributions are consistent with the ideal peptide plane structure of Fig 4, to the extent that any (minor) deviation could be attributed to a thermal fluctuation.

#### C*β* distributions in Anton simulations

**Fig 9:**
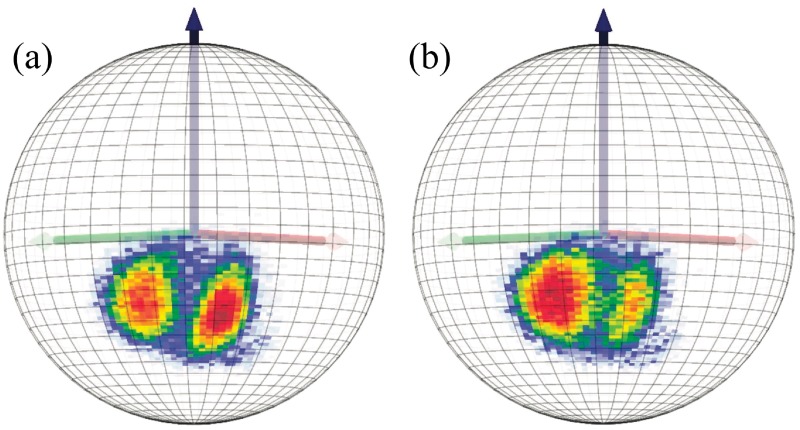
Statistical distribution of side chain C*β*(*i*) atoms as seen from the position of C*α*(*i*) atoms along the *Anton* trajectory; a) villin and b) ww-domain.

In Fig 9 we show the C*β* density distributions of Fig 6, along the *Anton* trajectories. In each case the distributions have a support which is very similar to that in Fig 6; the villin distribution has a higher population in the vicinity of the *α*-helical region of Fig 6 and the ww-domain has a higher population in the vicinity of the *β*-stranded region, as expected.

### Reconstruction of side chain C*β*(*i*) coordinates

#### Comparison of $\vec{C\alpha C\beta }$ direction with Frenet frame based reconstructions

**Fig 10:**
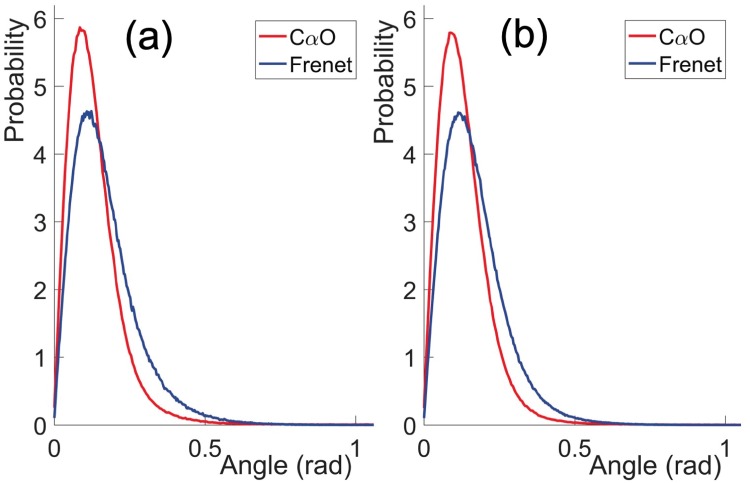
Comparison of the angle between the $\vec{C\alpha C\beta }$ directions in original *Anton* data and reconstructed data using the present C*α*O based *Statistical Method* and the Frenet frames based method of [22]. a) for the villin data and b) for the ww-domain data.

In [22] a Frenet frame based method has been developed to reconstruct the positions of atoms along a protein chain, from the knowledge of the C*α* atom positions. The present *Statistical Method* builds on that method. Thus, by comparing the results from these two methods we obtain an understanding how the knowledge of O atom positions influences the quality of reconstruction. For this we consider the vectors $\vec{C\alpha C\beta }$ that point from the C*α* atom to the ensuing C*β* atom. We evaluate these vectors from *Anton* simulations, and we reconstruct them using the two methods. We then compute the statistical (probability) distributions of the angles between the *Anton* vectors and the reconstructed vectors. The results are shown in Fig 10.

We find that in both methods, the predicted C*β* positions are extremely close to the original *Anton* results: The peaks of all four distributions in Fig 10 are very strongly peaked in the range of 0.08—0.11 radians; the values are slightly larger in the Frenet frames based reconstruction. With the C*α*-C*β* average covalent bond length ∼1.55 Å this corresponds to a distance in the range of 0.12—0.15 Å, which is hardly observable with presently available atomic level x-ray spectroscopy. For example, in our pool of ultra high resolution PDB structures, the Debye-Waller B-factor fluctuation distances all have values that are larger than 0.15 Å [22].

We observe from Fig 10 that the present, *Statistical Method* reconstructed C*β* atoms are systematically slightly closer to the C*β* atoms that are reconstructed in the Frenet frame based method. Thus, the inclusion of the peptide plane O atoms does appear to improve the reconstruction precision.

#### Comparison of $\vec{C\alpha C\beta }$ direction with ScwrIPP and Remo reconstruction

**Fig 11:**
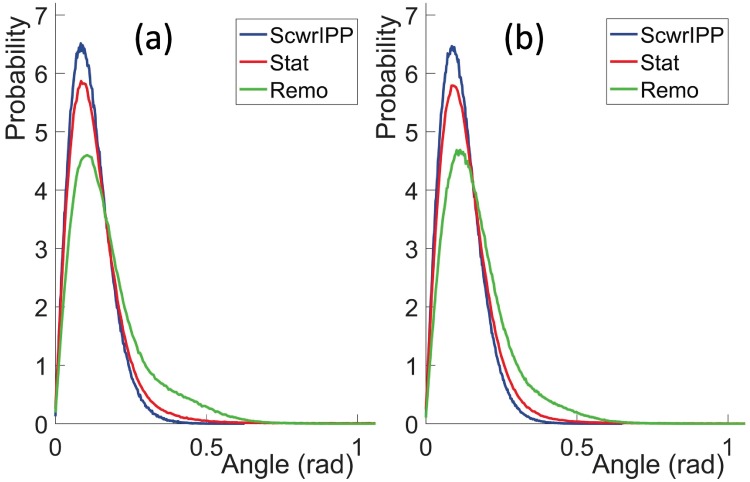
Comparison of the angle between the $\vec{C\alpha C\beta }$ directions in original *Anton* data and reconstructed data using the present *Statistical Method* (Stat), *ScwrIPP* and *Remo*. a) for the villin data and b) for the ww-domain data.

In Fig 11 we compare the $\vec{C\alpha C\beta }$ angles between the present *Statistical Method* and *ScrwIPP*. For comparison, we also compare with results from the *Remo* reconstruction program. In that case the only input is the C*α* positions of the *Anton* trajectory, the O(*i*) positions are determined by the program. The results shown in Fig 11 display the statistical (probability) distributions of the angles between the *Anton* vectors $\vec{C\alpha C\beta }$ and the reconstructed vectors $\vec{C\alpha C\beta }$. The results are very good in all three methods, those obtained with *ScwrIPP* are slightly better than those obtained with *Statistical Method*. The comparison to *Remo* reconstruction confirms that the inclusion of O atom positions leads to a visible and systematic improvement in the precision.

#### Statistical distributions of $\vec{O(i)C\beta (i)}$ on the two-sphere

**Fig 12:**
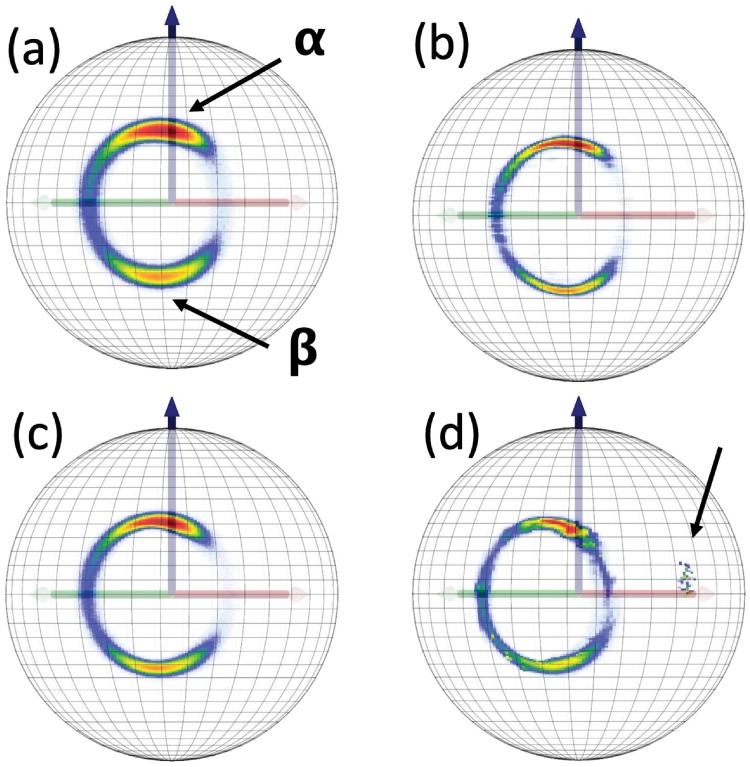
Statistical distribution of villin C*β*(*i*) atoms, when viewed from the position of O(*i*) atoms in C*α*O frames. a) Original *Anton* data, b) *Statistical Method* reconstructed data c) *ScwrIPP* reconstructed data and d) *Remo* reconstructed data. In a) we have identified the *α*-helical and *β*-stranded regions. In d) we have pointed out a dislocated region in *Remo* reconstruction.

**Fig 13:**
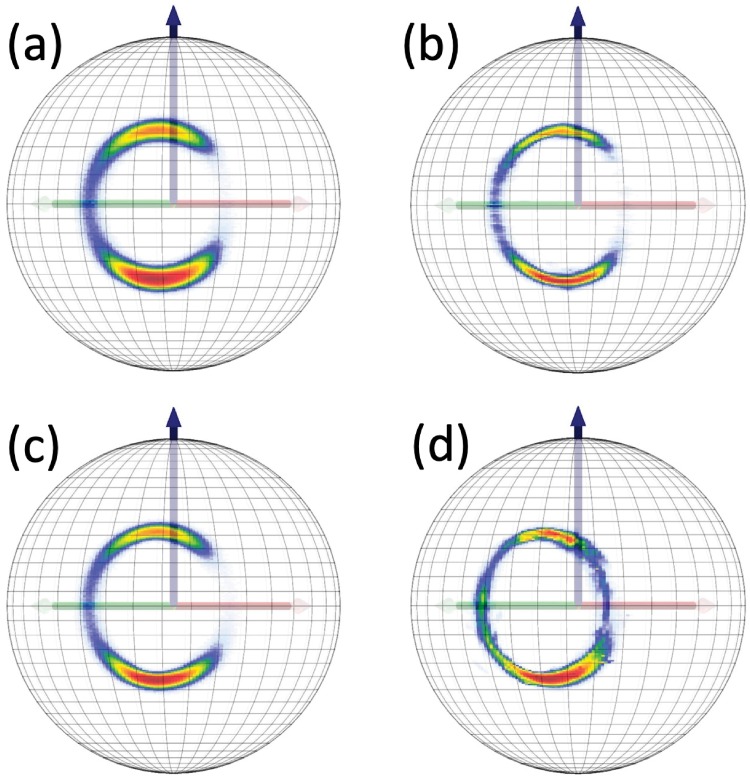
Statistical distribution of ww-domain C*β*(*i*) atoms, when viewed from the position of O(*i*) atoms in C*α*O frames. a) Original *Anton* data, b) *Statistical Method* reconstructed data c) *ScwrIPP* reconstructed data and d) *Remo* reconstructed data.

In Figs 12 and 13 we show various statistical distributions of C*β*(*i*) atoms, as they are seen in the C*α*O frames on the surface of a O(*i*) centered two-sphere. We show the results for the original *Anton* data, and the results from *Statistical Method*, *ScwrIPP* and *Remo* reconstructions. The *α*-helical and *β*-stranded regions are clearly identifiable in all distributions. We observe that *Statistical method* and *ScwrIPP* reproduce properly the original villin and ww-domain distributions. But in the case of *Remo* the distributions shows fragmentation and are also slightly, but systematically, shifted to the left, both in the case of villin and ww-domain. There is also an apparent small dislocated region in the villin distribution of *Remo*, pointed out in Fig 12d).

#### Comparison of $\vec{O(i)C\beta (i)}$ direction with ScwrIPP and Remo reconstruction

**Fig 14:**
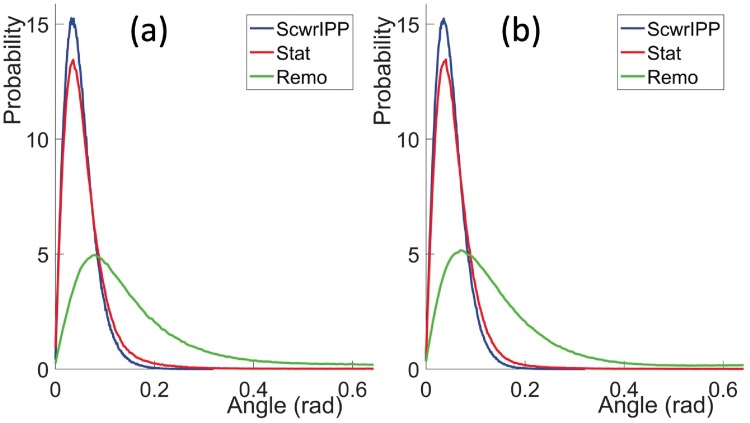
Statistical probability distributions for the angular difference between the *Anton* trajectory vectors $\vec{OC\beta }$ and the reconstructed vectors in the *Statistical Method*, *ScwrIPP* and *Remo*. a) villin, b) ww-domain.

In Fig 14 we compare the deviations in the statistical (probability) distributions for the reconstructed $\vec{O(i)C\beta (i)}$ angles in the case of *Statistical Method*, *ScwrIPP* and *Remo*. The reference structures are the $\vec{O(i)C\beta (i)}$ angles along the *Anton* trajectories, for villin and ww-domain. The deviations for *Statistical Method* and *ScwrIPP* are again very small and strongly peaked, at around 0.04 radians; for *Remo* the deviation is also small, but visibly less so. The distance between the C*α*(*i*) and the O(*i*) atom is around 2.40 Å. Thus, a peak at 0.04 radians in angular deviation corresponds to a distance around 0.05 Å. This is hardly observable with present day x-ray protein crystallography techniques.

#### Statistical distributions of $\vec{O(i)C\beta (i+1)}$ on a two-sphere

**Fig 15:**
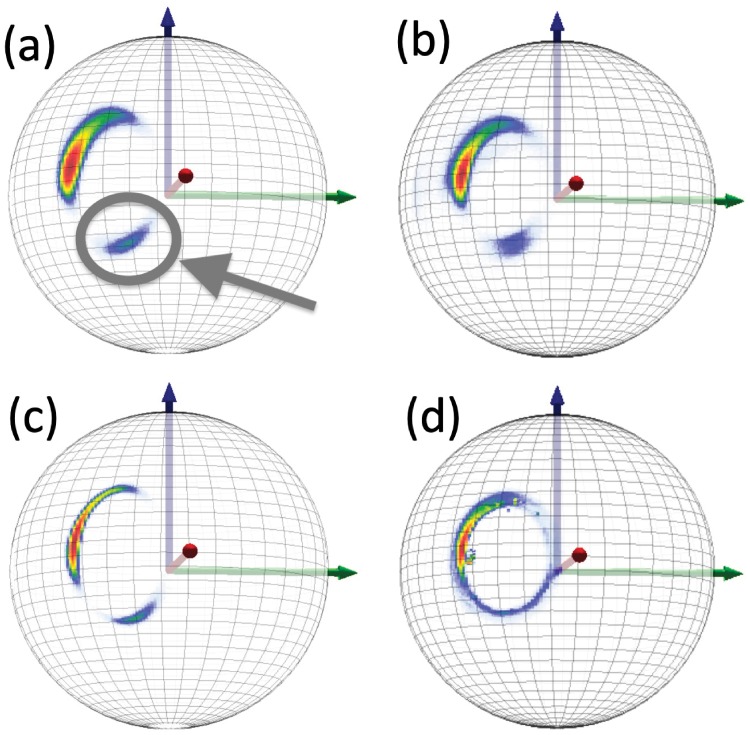
Statistical distribution of villin C*β*(*i* + 1) atoms viewed from position of O(*i*) atoms in C*α*O frames. a) Original *Anton* data b) *Statistical Method* c) *ScwrIPP* d) *Remo*. Note the isolated region that has been identified in Fig a.

**Fig 16:**
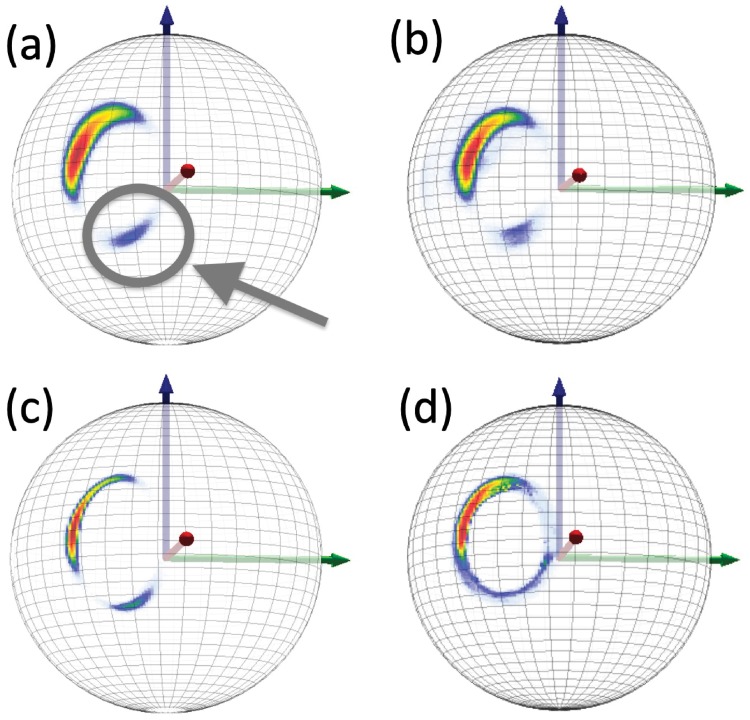
Statistical distribution of ww-domain C*β*(*i* + 1) atoms viewed from position of O(*i*) atoms in C*α*O frames. a) Original *Anton* data b) *Statistical Method* c) *ScwrIPP* d) *Remo* Note the isolated region that has been identified in Fig a.

In Figs 15 and 16 we compare the distributions of C*β*(*i* + 1) atoms, as they are seen on the C*α*O frames at the surface of O(*i*) centered two-spheres, for villin and ww-domain. We show the results for the original Anton data, and from *Statistical Method*, *ScwrIPP* and *Remo* reconstructions. We observe that the *Statistical Method* and *ScwrIPP* reconstructions are remarkably close to the original *Anton* distributions. The *Remo* reconstruction shown in the Fig 16d) displays more dispersion than the other two, shown in Fig 16b) and 16c).

#### Comparison of the C*β*(*i*)-O(*i*)-C*β*(*i* + 1) angle

**Fig 17:**
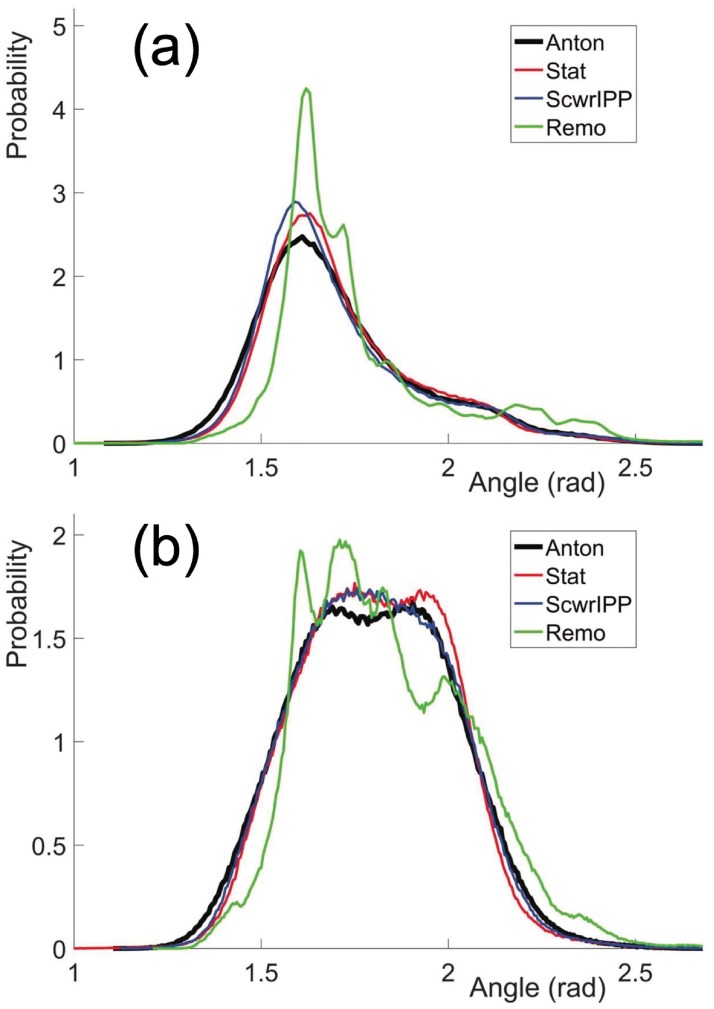
Statistical distribution of the angle ∠*Cβ*(*i*)O(*i*)C*β*(*i* + 1) for original *Anton* data and its reconstruction using *Statistical Method*, *ScwrIPP* and *Remo*. a) for villin, b) for ww-domain.

We combine the results in Figs 12 and 15, respectively in Figs 13 and 16 into analysis of the statistical distributions of the angle ∠*Cβ*(*i*)O(*i*)C*β*(*i* + 1) between the directions of the vectors $\vec{O(i)C\beta (i)}$ and $\vec{O(i)C\beta (i+1)}$. This measures the quality in the reconstruction of the entire C*α*-C*β*-peptide plane complex. In Fig 17 we show the results for both villin and ww-domain, in the four cases of original *Anton*, and *Statistical Method*, *ScwrIPP* and *Remo* reconstruction.

A comparison shows that the *Statistical Method* provides slight improvement over *ScwrIPP*. In the case of villin the peak in the *Statistical Method* is slightly closer to the original *Anton* peak, and in the case of ww-domain *Statistical Method* succeeds to reproduce the double peak profile of *Anton*. The difference between these two methods and *Remo* reconstruction is apparent, the latter displays visibly larger deviations from the original *Anton* data. This confirms that the knowledge of the O(*i*) atom positions brings about clear improvement in the reconstruction.

#### Isolated region in C*β*(*i* + 1) distributions

**Fig 18:**
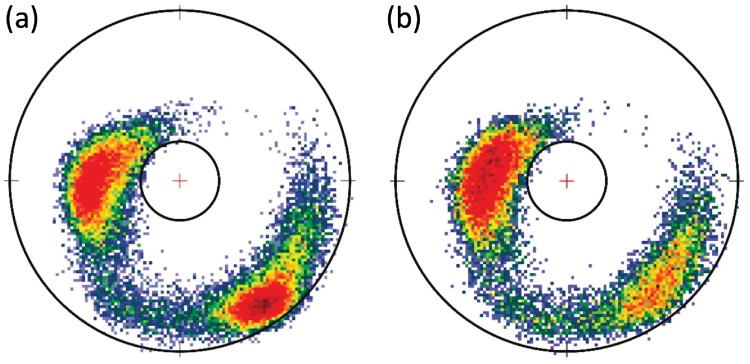
Statistical distribution of the (*κ*, *τ*) values that correspond to the isolated regions marked in Figs 15 and 16a). In Fig a) villin and in b) ww-domain.

In both Figs 15 and 16a), 16b) and 16c) we observe a very similar isolated region; in Fig d) this region becomes connected. In Fig 18 we show the locations of the corresponding regions on the stereographically projected two-sphere of Fig 5. The Fig 18 show that the isolated regions correspond to residues that are positioned either in the left-handed *α*-helical region or in a region that is the mirror image of the *β*-stranded region *i.e*. in both cases the torsion angle has become shifted by *τ* ∼ *π* with respect to the *τ*-values in the conventional *α*-helical and *β*-stranded regions. Note that the *π*-shifted *β*-stranded region is very rare among the crystallographic structures shown in Fig 5 which proposes that it might correspond to a higher energy, intermediate dynamical transition state.

## Discussion

We have investigated dynamical proteins using results from all-atom molecular dynamics simulation, with *Anton* supercomputer. In particular, we have inspected the very long time period *Anton* simulation trajectories of villin and ww-domain. We have found that the C*β* positions along these trajectories can be accurately reconstructed, solely from the knowledge of the backbone C*α* and O atom dynamics in combination with a statistical analysis of *static* crystallographic PDB structures.

We have compared the results that we obtain from our *Statistical Method* reconstruction, with publicly available all-atom reconstruction programs *Remo* and *Scwrl4*. *Remo* starts from the C*α* trace, then reconstructs the peptide planes using elaborate optimization protocols. It then proceeds with *Scwrl* to add the side chain atoms for the full all-atom structure. *Scwrl4* can also be used in a stand-alone mode, and for this we have enhanced it using the knowledge of the C*α* and O atom coordinates along the *Anton* trajectories, with N, C and H in ideal peptide plane positions. Both *Remo* and *Scwrlt4* need to reconstruct the entire all-atom structure, to evaluate the C*β* coordinates. This provides added precision to the reconstruction, as the removal of higher level steric clashes serves as a constraint. On the other hand, the present *Statistical Method* places the C*β* atoms with no regard to higher level side chain structures, or potential steric clashes between side chains and peptide planes. Only the backbone C*α* and O atoms time evolution is employed in the reconstruction. Nevertheless, the results that we get using the *Statistical Method* are a clear improvement over those obtained with *Remo*, and are fully comparable with those obtained by our enhanced *Scwrl4*. Thus, at the present level of scrutiny, side chain dynamics and in particular that of C*β* atoms, appears to be fully slaved to that of the backbone C*α* and O atom dynamics. The scrutiny of the higher level side chain atoms does not seem to bring about much improvement in reconstructing the C*β* dynamics.

All the three methods that we have employed are, by their construction, designed to reconstruct static low temperature crystallographic protein structures. They are not intended to model the all-atom structure of a dynamical and biologically active protein at physiological temperatures. Nevertheless, each of them succeeds in the reconstruction of the C*β* dynamics in *Anton* simulations, with an impressive precision. We find this to be remarkable and very surprising. It motivates the development of coarse grained approaches to model large time scale protein dynamics, in terms of reduced coordinates that describe the dynamics of C*α*, O and C*β* atoms only.
